# Advances in Bioactive Peptides: Innovations in Food Processing, Functional Foods, and Health

**DOI:** 10.3390/foods15213938

**Published:** 2026-11-03

**Authors:** Mohammad Zarei

**Affiliations:** Department of Nutrition and Food Science, California State Polytechnic University, Pomona, CA 91768, USA; mzarei@cpp.edu

## 1. Introduction

Food proteins carry short amino acid sequences that stay inactive while they remain part of the parent protein. Enzymatic hydrolysis, microbial fermentation, and gastrointestinal digestion release these sequences, and once free they can lower blood pressure, slow starch digestion, scavenge free radicals, protect the intestinal lining, and stabilize emulsions. The number of reported sequences has grown quickly over the past three decades, and public databases now hold thousands of them [1,2].

This pace of discovery has created a problem of its own. Most reported peptides rely on in vitro assays. Fewer have been tested on animals, fewer still in humans, and only a small number have reached the market as ingredients supported by an approved health claim. The reasons are well documented. Peptides are degraded during digestion, transport across the intestinal barrier is limited, activity is often lost when a peptide is placed in a real food, production at scale is costly, and bitterness can block consumer acceptance [2,3,4]. Structure and activity are still linked case by case rather than through general rules, which keeps rational design out of reach for most targets [5].

This Special Issue, “Advances in Bioactive Peptides: Innovations in Food Processing, Functional Foods, and Health”, was launched to gather work that moves past the discovery step. Six contributions were accepted after peer review. They cover peptide preparation from an underused oilseed by-product, computational screening closed by laboratory confirmation, mechanism described at the level of the whole proteome, two animal studies with disease endpoints, and one study that places peptide fractions inside a working food system. Read together, they follow the path a peptide has to travel from a protein source to a product.

## 2. Overview of the Published Articles

Guo et al. worked with *Elaeagnus mollis* oilseed meal, a protein-rich residue left after oil extraction (Contribution 1). Fourteen proteases were compared before an acidic protease was selected and the hydrolysis conditions were optimized. The resulting hydrolysate inhibited α-glucosidase with an IC50 of 9.11 mg/mL, against an acarbose control at 0.0085 mg/mL, and showed no detectable cytotoxicity toward HEK-293T cells between 0.1 and 12.0 mg/mL. Ultrafiltration gave four fractions, of which the 3–10 kDa fraction was the most active and held much of its activity under acidic pH, moderate temperature, salt, and simulated gastrointestinal digestion. The study shows how a low-value processing residue can be converted into a candidate ingredient for blood glucose management, and the enzyme screening step is a useful reminder that protease choice sets the ceiling for everything that follows.

Yang et al. took a computational route (Contribution 2). Three major royal jelly proteins were digested in silico with sixteen enzyme systems, which produced 1411 unique peptides. Screening on predicted bioactivity, toxicity, water solubility, and ADMET properties narrowed the list to 27 candidates, all of which docked to ACE with stronger predicted binding than captopril. The four best-ranked candidates were synthesized, and two, PYPDWSFAK and RPYPDWSF, were confirmed as ACE inhibitors with IC50 values of 110 and 204 μmol/L. Docking placed PYPDWSFAK in the ACE active pocket, hydrogen bonded to S1 residues and coordinated directly to the catalytic zinc. In endothelial cells the peptide was non-cytotoxic, suppressed angiotensin II-induced migration, and raised nitric oxide while lowering endothelin-1 after angiotensin II challenge; in a separate hydrogen peroxide model it partly recovered superoxide dismutase and glutathione peroxidase activity. The design is worth noting because the screening was closed with laboratory work at both the enzyme and the cell level rather than stopping at the docking score.

Mohamed et al. used quantitative proteomics to ask what a peptide does to a cell rather than to a single enzyme (Contribution 3). Data-independent acquisition was applied to an insulin-resistant HepG2 model and to cells treated with a camel milk-derived peptide. Insulin resistance was associated with broad reorganization of protein networks tied to redox regulation, endoplasmic reticulum protein processing, mitochondrial metabolism, lysosomal function, and extracellular matrix components. Gene ontology, pathway, protein domain, and subcellular localization analyses pointed to disruption of organelle-level architecture rather than to a single perturbed pathway, and peptide treatment shifted part of this landscape. Camel milk has been studied for antidiabetic effects for some time [6,7], and this contribution moves the discussion from whole milk or whole protein to a defined sequence and a measurable proteomic response.

Xu et al. gave mung bean peptides to mice before and throughout colitis induced by dextran sulfate sodium (Contribution 4). The preparation was first characterized by amino acid composition, molecular weight distribution, and peptide sequences. Peptide administration preserved colon length and reduced histological damage and the disease activity index. It increased the expression of the tight junction proteins ZO-1 and claudin-1 in colonic tissue, lowered serum IFN-γ, IL-6, and IL-1β, and changed the structure of the gut microbiota. The design is preventive rather than therapeutic, and the study connects a barrier effect, a systemic inflammatory effect, and a microbial effect within one animal model, which is more informative than any of the three alone.

Tocharus and Sutheerawattananonda examined sericin-derived oligopeptides from yellow silk cocoons in rats made hypertensive with L-NAME (Contribution 5). Separate preventive and therapeutic experiments each used nine groups, covering three peptide doses, enalapril as a drug comparator, and soy protein isolate and bovine serum albumin as protein comparators. Doses of 100 and 200 mg per kg body weight lowered systolic blood pressure without changing heart rate, the 200 mg dose came closest to the enalapril group, and vascular relaxation improved with dose. The two protein controls are a design feature that other groups should copy, and here they mattered: by the authors’ own assessment, soy protein isolate lowered blood pressure roughly as much as the peptides did, a result that could not have been seen without it.

Yay et al. moved the question into a food system (Contribution 6). Potato protein hydrolysate produced with trypsin was first screened in small-scale emulsions, where the unfractionated hydrolysate alone gave unstable emulsions and combining it with Tween 20 or DATEM improved physical stability. It was then used unfractionated and as four size fractions, each combined with one of the two surfactants, in 5 wt% fish-oil-in-water emulsions. Fractions below 10 kDa gave the smallest droplets with DATEM, between 0.352 and 0.764 μm, whereas with Tween 20 the smallest droplets came from the unfractionated hydrolysate at 1.051 ± 0.015 μm. Oxidative stability was best for the 5–10 kDa fraction combined with Tween 20. The fraction that performed best on droplet size was therefore not the fraction that performed best on oxidation, which is the kind of trade-off that appears only once peptides are asked to work inside a product.

## 3. Themes Running Through the Collection

### 3.1. Side Streams as Protein Sources

Half of the contributions start from material that is not the main product of its process. *Elaeagnus mollis* meal is left after oil extraction, potato protein is recovered from starch processing, and sericin is removed from silk during degumming. This is not a coincidence of sampling. Peptide production is one of the few routes that gives a low-value stream a high-value destination without requiring a new crop or a new supply chain [1]. It also changes the economics of the ingredient, because the protein has already been paid for by the primary product.

### 3.2. Computation as a Filter Rather than a Substitute

Contribution 2 shows what in silico screening is good for. It reduced 1411 candidate sequences to 27, and then to two confirmed inhibitors, which would be impractical by synthesis and assay alone. It also shows the limit. A docking rank is a hypothesis, and in that study the peptide with the best predicted binding, FDVDRW, was outperformed in the enzyme assay by two sequences ranked below it. Contribution 3 uses computation at the other end of the workflow, not to find a peptide but to read the cellular response to one. Both uses are needed, and neither replaces the bench.

### 3.3. Mechanism Described at Several Levels

Across the six papers, the mechanism is described at the level of enzyme kinetics and binding, cell function, tissue barrier proteins, vascular response, systemic inflammation, and microbial community structure. What no study in this collection does, and what very few studies anywhere do, is follow one defined sequence through all of these levels in the same system. That gap is a main reason why it remains hard to explain why a peptide that is potent against an isolated enzyme often disappoints in a feeding trial.

### 3.4. The Matrix and the Dose

Contribution 6 is a reminder that a peptide fraction can be bioactive and still fail as an ingredient. Molecular weight distribution and the choice of co-surfactant decided whether the emulsion held together, and the best fraction for physical stability was not the best fraction for oxidative stability. A similar tension appears on the health side. The doses in Contribution 5 are expressed per kilogram of body weight in rats and cannot be read directly as a serving size for people. Bringing a peptide into a food means solving both problems at the same time [4].

## 4. Gaps That Remain

Human evidence. All of the in vivo work in this collection was carried out in rodents. Rodent studies are the right place to establish plausibility, but the field will not move into food labels until controlled human studies are run at intakes that a person could realistically consume in a normal food format.

Bioavailability and stability. An IC50 measured against an isolated enzyme says little about what reaches the bloodstream. Contribution 1 is the one study here that carried its active fraction through simulated gastric and intestinal digestion, and the result was worth having: activity fell but did not disappear. Digestion stability, transport across intestinal cell models, and plasma persistence should be reported as routinely as inhibition data, and encapsulation should be evaluated as part of the peptide rather than as an afterthought [8].

Reporting that allows comparison. Inhibition values in this field are reported in mg/mL by some groups and in μmol/L by others, positive controls differ, and the degree of hydrolysis and enzyme source are not always stated. These choices are individually reasonable and collectively make it very hard to rank peptides across studies. Reporting molar values alongside mass values, naming the enzyme preparation and its activity units, and including a common positive control would cost authors little and would help every reader.

Cost and scale. Ultrafiltration, chromatographic fractionation, and synthesis are usual steps in the discovery workflow and unusual steps in a food plant. Papers that report yield per kilogram of starting material, and the fraction of activity retained after each unit operation, are more useful to industry than papers that report only the potency of a purified sequence.

Taste and acceptance. Hydrolysis generates bitterness along with activity, and the two often increase together. Sensory evaluation is still missing from most peptide papers, including several strong ones [3].

## 5. Future Directions

Machine learning will keep expanding the candidate space, and the value of these models will depend on the quality and consistency of the training data rather than on the architecture. Curated datasets with standardized assay conditions, and the publication of sequences that were predicted to be active and were not, would improve prediction more than any single new model.

Multi-omics work of the kind shown in Contribution 3 should be pushed toward causality. Showing that a proteomic or microbial profile changes after treatment is a starting point; targeted knockdown, gnotobiotic models, or transfer experiments are needed to show that change carries the effect.

Defined mixtures deserve as much attention as single sequences. Contributions 1, 4, 5, and 6 all worked with peptide mixtures, and in each case the mixture was the practical unit of use. A mixture that is reproducible, characterized, and stable may be a more realistic ingredient than a single purified peptide, and the field would benefit from methods that specify mixtures well enough to be regulated.

Finally, sustainability claims attached to by-product valorization should be measured rather than asserted. Life cycle assessment and techno-economic analysis belong alongside the bioactivity data when a peptide is presented as a circular economy solution [1].

## 6. Conclusions

The six contributions in this Special Issue move along the same line from a protein source to a health or technological function, and each one stops at a different point on that line. Taken together they show a field that is confident about discovery and still uneven about translation. The most useful future studies will not be the ones that report another potent sequence, but the ones that carry a defined preparation through digestion, into a food, and into a controlled human study with a dose that a person would actually eat.

I thank all authors who submitted their work, the reviewers whose careful reading shaped the accepted papers, and the editorial office of *Foods* for its support throughout the life of this Special Issue.
